# Characterizing relationship between chemicals and in vitro bioactivities of teas made by six typical processing methods using a single Camellia sinensis cultivar, Meizhan

**DOI:** 10.1080/21655979.2021.1903237

**Published:** 2021-04-27

**Authors:** Guanhua Xie, Jingna Yan, Anxia Lu, Jirui Kun, Bei Wang, Chengda Song, Huarong Tong, Qing Meng

**Affiliations:** College of Food Science, Southwest University, Chongqing, China

**Keywords:** Camellia sinensis, teas, processing, bioactivity, relationships

## Abstract

Processing method is considered as a major factor that affects biotransformation of phytochemicals in tea and leads to diverse flavor and bioactivity of tea. In the present work, six typical tea manufacturing processings were employed to compare the effect on chemical composition of teas through using leaves of the single tea cultivar – – Camellia sinensis var. Meizhan. And in vitro antioxidant activity, inhibition against α-glucosidase and three lipid metabolism enzymes of these teas were also investigated, the relationships among them were analyzed further. As fresh leaves were processed into six categories of teas, the content of total catechins (TCs) has decreased in varying degrees while theaflavins (TFs) has increased. The antioxidant capacity composite index (ACCI) from high to low were green tea, yellow tea, oolong tea, white tea, dark tea, and black tea with the range from 98.44 to 58.38, which dominated by the content of TCs. Furthermore, all categories of teas possessed an inhibition effect on the pancreatic lipase (PL), 3-hydroxy-3-methylglutaryl coenzyme A reductase (HMG-COA reductase), lecithin cholesterol acyltransferase (LCAT), and α-glucosidase. The inhibition rate of PL and α-glucosidase appears to be positively influenced by TFs content (r =0.863, r =0.857, p < 0.05) while that of LCAT showed significant positive correlations with the content of tea polyphonels (TPs) (r = 0.902, p < 0.01). These results provide a better understanding of the relationships between processing method and chemical components of tea. It is suggested that various tea categories possess potential healthy effects which could serve as promising nutritional supplements.

**Figure:**
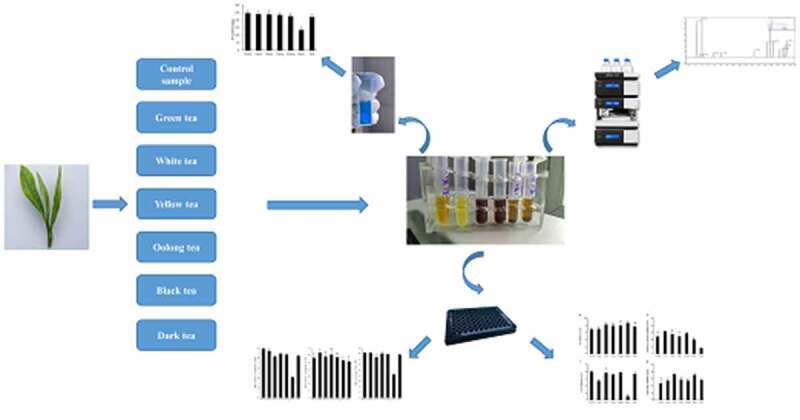

## Introduction

Tea is a long-lasting beverage worldwide only after water. After the harvest from a plant named Camellia sinensis, the fresh buds and leaves are made into numerous tea products. Based on the manufacturing methods and flavor of products, the types of tea could be classified as green tea (non-fermented), white tea (lightly fermented), yellow tea (partially fermented), oolong tea (semi-fermented), black tea (fully fermented), and dark tea (post-fermented) [1]. As known, tea possess unique flavor and potential function for healthy owing to rich in bioactivity materials, such as catechins, alkaloids, free amino acids, vitamins and others [2]. The manufacturing process dominate the aroma and taste characteristics of tea through change the proportion of chemical components. Usually, tea polyphonels (TPs) is considered as the typical materials in teas because it accounts for over 20% of the dry weight. Thereinto, catechins is the key element which including (-)-epigallocatechin gallate (EGCG), (-)-epigallocatechin (EGC), (-)-epicatechin gallate (ECG), and (-)-epicatechin (EC), etc. [3]. After fermentation during process, catechins could be converted into theaflavins (TFs) and thearubigins (TBs) [4].

Previous studies have shown that tea components could relieve metabolic syndrome through directly influencing the activity of key enzymes [5]. For example, green tea, black tea, and oolong tea could efficiently inhibit against α-glucosidase, a key enzyme in carbohydrate metabolism, and their inhibitory potency is mainly attributed to TPs [6]. Gallated catechins have shown better α-glucosidase inhibition effects than non-gallated catechins [7]. White tea, green tea, and black tea could reduce the glucose and cholesterol uptake, and possess different inhibition capacities against pancreatic lipase (PL) activity [8]. Additionally, pu-erh tea extraction could increase the content of high-density liptein cholesterol (HDL-C) and enhance the activity of lecithin cholesterol acyltransferase (LCAT), thereby possessed a significant blood effect of lipid-lowering [9,10]. The difference in health benefits among teas mainly due to the composition of bioactive components, especially the contents and types of catechins.

Recently, the tea science field has not only focused on the formation of tea flavor, but the bioactivity of tea has attracted lots of interest also [11,12]. Plenty of works have evaluated the chemical compounds and bioactivity of different categories of teas [13–15]. However, due to the differences in tea plant cultivar, processing methods, and growing conditions, these results were often inconsistent or even contrary. It is a limitation to reveal the relationship between chemical components and their bioactivity. Taken together, set a limit to cultivar and area would be the solution to these confusing issues.

Meizhan is one of the most widely cultivated varieties in China, with rich aroma components and widely processing suitability [16,17]. Aim to disclose the impact of tea processing method on phytochemicals of tea and explore the potential health effect of various teas, meanwhile, minimize the influence of material differences. In the present work, the fresh leaves of Meizhan under identical conditions were plucked and processed into six categories of teas by typical manufacturing processes. The chemical components of the fresh leaves and six categories of teas were analyzed and compared in this study. Their antioxidant capacity composite index (ACCI), the effect on regulation of lipid metabolism enzymes and α-glucosidase inhibition were also investigated. Furthermore, the relationships between chemicals and in vitro bioactivities were analyzed further.

## Materials and methods

### Chemicals

4-Methylumbelliferyl oleate (4-MU oleate) was purchased from Titan Biotechnology Co. (Shanghai, China), PL (EC 3.1.1.3) and 3-hydroxy-3-methylglutaryl coenzyme A reductase (HMG-CoA reductase, EC 1.1.1.34) was obtained from Bositai Biotechnology Co., (Chongqing, China). LCAT (EC 2.3.1.43) were purchased from Feiyu Biotechnology Co. (Jiangsu, China). α-glucosidase (EC 3.2.1.20) was obtained from Shanghai Ryon Biological Technology Co. (Shanghai, China). Standards of gallic acid (GA), caffeine (CAF), methanol, acetonitrile, and acetic acid of HPLC grade were obtained from Sigma Chemical Co. (Missouri, USA). 2,2-diphenyl-1-picryl-hydrazyl (DPPH), 2,2’-azinobis (3-ethylbenzothiazoline-6-sulfonic acid) diammonium salt (ABTS), 6-hydroxy-2,5,7,8-tetramethylchroman-2-carboxylic acid (Trolox), and fluorescein disodium were obtained from Amida Biotechnology Co. (Chongqing, China). All the standards of individual catechins, 4-nitrophenyl b-D-glucuronide (PNPG) and all other chemicals of analytical grade were purchased from Aladdin Biotechnology Co. (Shanghai, China).

### Preparation and extraction of tea samples

**Figure 1.**
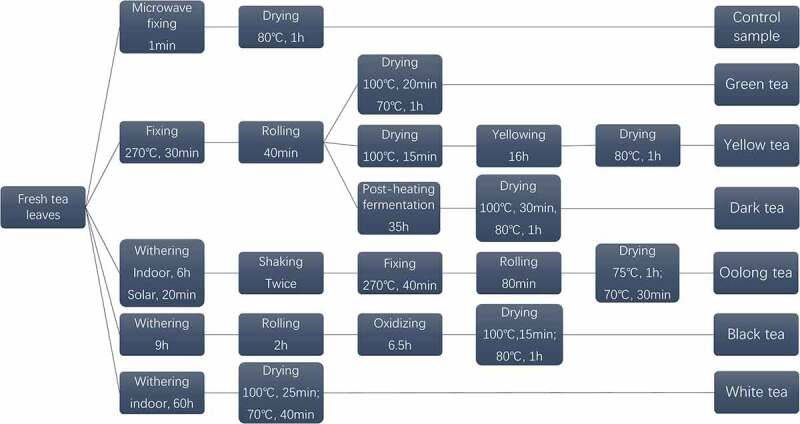
Flow chart illustrating the different stages in the manufacture of six teas

Fresh tea leaves of Meizhan were harvested on 12 September 2018 from Nanrun Tea Co., Ltd (Banan district, 106°54ʹ N, 29°40ʹ E, Chongqing, China). All of the fresh leaves were then processed into six categories of teas according to six processing methods, while the control group was processed through fixing the fresh tea leaves directly by microwave (Figure 1). Through sensory evaluation by GB/T 23776–2018. And then, all samples were ground into a powder. 0.2 g of tea powder were extracted twice in 5 mL of 70% methanol at 70°C for 10 min. After centrifugation, the supernatant was merged and used for the determination of the TPs content and HPLC analysis. 3 g of tea powder were steeped in 100 mL of boiling ultrapure water for 45 min. After filtration, the extract was stored at 4°C for determination of antioxidant activity and regulation effect on the relevant enzymes.

### Analysis of the main nonvolatile composition

#### Determination of total TPs content

The total TPs content of tea extracts was determined using the Folin–Ciocalteu method with slight modifications and was expressed as gallic acid equivalents using gallic acid (GA) as standard [18]. Breifly, 1 mL of methanol extracts was diluted to 100 mL. 1 mL diluted solution was mixed with 5 mL of Folin–Ciocalteu reagent and reacted for 8 min. And then, added 4 mL of 75% sodium carbonate into the mixture and kept at room temperature for 60 min. Finally, the absorbance was measured by a microplate reader at a wavelength of 765 nm.

#### Determination of catechin components, caffeine, and theaflavins by ultra high-performance liquid chromatography

The contents of individual catechins, caffeine, and theaflavins in tea extracts were determined according to the UPLC method with some modifications [19]. In short, 5 µL of tea extracts were injected into a Thermo HPLC UltiMate 3000 system coupled with a Thermo Diode Array Detector (DAD), and the separation was performed on a Ascentis®RP-Amide column (5 µm, 25 cm x 4.6 mm) according to the following: injection volume, 5 μL; column temperature, 35°C; UV detection wavelength, 278 nm. Mobile phase A (water containing 0.2% acetic acid) and mobile phase B (100% acetonitrile); the mobile phase flow, 1 mL/min; Linear elution of mobile was as follows: B began with 15% from 0 to 20 min, to 35% at 25 min, to 45% at 40 min, to 15% at 45 min and kept at 15% for 3 min.

### Measurement of antioxidant activity in vitro

#### DPPH radical scavenging activity

The DPPH radical scavenging rate of the tea extracts was measured according to the method described by Cheng et al. [20]. Tea extracts were diluted with ultrapure water the DPPH radical stock solution was prepared in 50% ethanol (0.625 mM) monthly and kept at 4°C in dark. The diluted tea extracts (50 µL) were mixed with DPPH radical stock solution (150 µL) or 50% ethanol solution (150 µL), respectively. The absorbance of the resulting mixture was measured against a blank sample at 515 nm after 40 min in the dark at 25°C. The DPPH radical scavenging rate was calculated as follows:

DPPH radical scavenging rate (%) = [1 -$\;\frac{A_{samp}-A_{samp\&ethanol}}{A_{ethanol}}$]x 100% (1)

#### ABTS radical scavenging activity

The ABTS free radical scavenging activity of the infusions was determined by the method of Thaipong [21] with slight modifications. The working solution was freshly prepared by mixing equal ABTS+ solution (7.4 mmol/L) and potassium persulfate solution (2.6 mmol/L) and reacted for 12 h at 25°C in the dark. 1 mL of infusion was then diluted by 15 mL of methanol to obtain an absorbance of 1.1 ± 0.02 units at 734 nm using the microplate reader (SpectraMax M5, Molecular Devices, Sunnyvale, CA, USA). The diluted tea extracts (25 µL) was mixed with the diluted infusion (200 µL) or the methanol solution (200 µL), respectively. The absorbance of the resulting mixture was measured against a blank sample at 734 nm after 6 min in the dark at 25°C. The ABTS radical scavenging rate was calculated according to the following equation

ABTS radical scavenging rate (%) = [1 -$\;\frac{A_{samp}-A_{samp\&methanol}}{A_{methanol}}$] x 100% (2)

#### Oxygen radical absorption capacity (ORAC)

The oxygen radical absorption capacity of different tea extracts was measured according to the method of Kelly [22]. Briefly, 20 µL of blank, Trolox standard, or tea extracts in 75 mmol/L phosphate buffer (PPB, pH 7.4) was reacted with 200 µL of 0.96 µmol/L fluorescein at 37°C for 20 min, respectively. Followed by adding 20 µL of 119 mmol/L ABAP, the absorbance was measured at inserted into a microplate reader at 37°C. Fluorescence was collected at 538 nm on excitation at 485 nm with 2.5 nm slit widths, taking measurements from each sample at 270 s intervals for 2.5 h. The net area under the curve (AUC) of the standards and samples was calculated as follows:

Net-AUC = $AUC_{sample}-AUC_{blank}$ (3)

Final ORAC values are calculated using the regression equation between Trolox concentration and the Net-AUC and are expressed as micromole Trolox equivalents per gram for solid samples.

#### Calculation of Antioxidant capacity composite index (ACCI)

ACCI [23] was determined by assigning all assays an equal weight, assigning an index value of 100 to the best score for each assay, and then calculating an index score for all other samples within the assay as follows:

antioxidant index score = [(sample score/best score) × 100] (4)

The average of all three assays for each tea sample was then taken for the ACCI.

### Determination of lipid metabolism enzymes activity in vitro

#### Pancreatic lipase (PL) inhibition activity

The PL inhibitory activity was measured using 4-MU oleate as a substrate [24]. In brief, 50 µL of 4-MU oleate solution (50 U/mL) was added to the buffer (pH 8.0) which dissoved 25 µL tea extracts prepared above and 25 µL Pancreatic lipase solution (0.1 mmol/L) and reacted for 30 min at 25°C. Then, to terminate by 0.2 mL of 0.1 M sodium citrate (pH 4.2). The absorbance was measured at an excitation wavelength of 355 nm and an emission wavelength of 460 nm. The inhibition rate of PL activity was calculated as follows:

The inhibition rate of PL activity (%) = $\left[1-\left(\frac{A_{sample}-A_{blank}}{A_{test}-A_{control}}\right)\right]$ x 100% (5)

#### 3-Hydroxy-3-Methylglutaryl Coenzyme A Reductase (HMG-CoA reductase) inhibition activity

The HMG-COA reductase inhibitory activity was quantified using an enzyme-linked immunosorbent assay (HMG-COA ELISA Kit). Tea extracts (25 µL), HMG-COA reductase solution (25 µL), and 100 µL of phosphate buffer (PBS) were successively added to a 2 mL test tube, while the blank sample was prepared without tea extracts. After incubated at 37°C for 15 min, the activity of HMG-COA reductase was quantified in a ELISA plate according to the manufacturer’s instructions.

#### Lecithin cholesterol acyltransferase (LCAT) inhibition activity

The activity of LCAT was quantified using an enzyme-linked immunosorbent assay (LCAT ELISA Kit). Tea extracts (25 µL), LCAT solution (25 µL), and PBS (100 µL) were successively added to a 2 mL test tube, while the blank sample was prepared without tea extracts. After incubated at 37°C for 15 min, the activity of LCAT was quantified in a ELISA plate according to the manufacturer’s instructions.

#### α-glucosidase inhibition activity

The α-glucosidase inhibitory activity of the tea extracts was determined according to the method described by Tan et al. with slight modifications [25]. In brief, 80 µL of tea extracts were mixed with 100 µL of 4 mM pNPG solution (dissolved in 0.1 M PBS, pH 6.8) and reacted with 20 µL of 25 U/mL enzyme solution at 37°C for 10 min. The absorbance of the reaction mixture was measured at 405 nm by microplate reader (SpectraMax M5, Molecular Devices, Sunnyvale, CA, USA). The inhibition rate of α-glucosidase ability was calculated as follows:

inhibition (%) = $\left[1-\left(\frac{A_{sample}-A_{blank}}{A_{test}-A_{control}}\right)\right]$ x 100% (6)

### Statistical analysis

All of the experimental results were expressed as mean ± standard deviation (SD) of at least three replications. Data were represented the mean triplicate analysis using One-way ANOVA with SPSS 18.0 statistical software. Statistical analysis of the data was conducted using Duncan’s test to evaluate significant differences between a pair of means (P < 0.05). Correlations (r) of data were evaluated using Pearson’s correlation analysis.

## Results

### Tea process design and sensory evaluation of the tea

To investigate the differences of the chemical components caused by manufacturing processes and evaluate their bioactivities. The principle of the experiment design is to optimize tea processing methods, meanwhile, to ensure the flavor and characteristic components of six tea samples can be maintained and distinguished from each other. In order to minimize the interference factors, the leaves from a single C. sinensis Meizhan cultivar were harvested at the same time. And these six categories of teas were designed and processed by an experienced tea processing expert according to the typical tea manufacturing processes. All tea products possessed typical sensory characteristics and consistent with the commonly accepted classification.

### Analysis of total TPs, catechins, caffeine, and theaflavins concentrations among tea types

Diverse manufacturing processes generate different types of tea which possess unique flavor through convert composition of chemicals in fresh leaves. To investigate the effect of processing methods on tea differentiation, we determined the major nonvolatile compounds of the tea samples. As shown in Table 1, it could be seen that the TPs content from high to low was green tea, white tea, yellow tea, oolong tea, dark tea, and black tea, which varies from 238 to 133 mg/g. TPs content in black tea was significantly lower than those in all the others owing to the effect of polyphenol oxidases and peroxidases during processing [26]. Catechins are major components in tea polyphenol, their types and contents are considered as the major factor to distinguish tea category from each other [27]. It could be partially oxidized to dimers, such as theasinensins and theaflavins through fermentation [28]. And which could continue to biotransform into thearubigins, theabrownine, and other poorly characterized highmolecular-weight polyphenol-complexes [29–31]. It is reasonable for the lowest content of black tea catechins. As a result of oxidation degree, catechins were preserved as monomeric forms in green tea. EGCG was the most abundant catechin in all the tested teas, especially in green tea and yellow tea. The content of EGC was second only to EGCG while it was not detected in black tea. Meanwhile, total TFs exhibited the highest content in black tea. It is logical that fully fermented black tea has a much higher conversion rate of catechins to theaflavins and thrubigins [32]. Among the four kinds of TFs monomers, theaflavin (TF) accumulated most abundantly in all teas except black tea while theaflavin-3,3’-digallate (TFDG) was the most abundant one in black tea. Interestingly, white tea had the highest content of TFs in partially fermented teas (yellow tea, oolong tea, and white tea) and dark tea showed the lowest of all. Furthermore, as another characteristic components of tea, there was no significant difference in the CAF level of all tested teas. It means procedure of fermentation would not affect the concentration of caffeine.

**Table 1.** The content (mg/g) of catechin components, caffeine, theaflavins, and total polyphenols in the control sample and six categories of teas

Tea	CAF	Catechin			Esters-catechin			Total Catechins	TF	TF-3-G	TF-3’-G	TFDG	Total TFs	Total TPs
		EGC	C	EC	EGCG	GCG	ECG							
Control	36.29 ± 1.70a	61.96 ± 4.39e	1.41 ± 0.05d	14.54 ± 0.86 f	99.30 ± 5.78 f	0.71 ± 0.04bc	19.15 ± 1.20d	198.40 ± 2.01 f	0.34 ± 0.04a	0.05 ± 0.00a	0.16 ± 0.01a	0.29 ± 0.02a	0.84 ± 0.02a	245.15 ± 13.31a
Green	37.31 ± 1.00a	49.77 ± 1.67d	1.08 ± 0.05 c	11.35 ± 0.35e	91.87 ± 4.06e	0.62 ± 0.03b	18.08 ± 0.72d	175.10 ± 6.95e	1.42 ± 0.10 c	0.30 ± 0.02 c	0.25 ± 0.01 c	0.37±0.02ab	2.34 ± 0.15b	238.20 ± 9.14a
White	36.80 ± 1.91a	23.81 ± 1.24b	0.96 ± 0.08b	4.81 ± 0.25b	61.54 ± 3.48b	1.52 ± 0.14e	14.09 ± 0.80b	107.97 ± 6.03b	3.43 ± 0.20 f	0.99 ± 0.08e	0.40 ± 0.02e	0.74 ± 0.04 c	5.55 ± 0.33d	236.36 ± 36.77a
Yellow	38.20 ± 0.36a	39.48 ± 0.43 c	1.04 ± 0.02bc	9.60 ± 0.09 cd	86.46 ± 1.10e	0.75 ± 0.03 c	18.44 ± 0.17d	159.16 ± 1.81d	1.24 ± 0.02bc	0.30 ± 0.01 c	0.28 ± 0.00d	0.39 ± 0.01b	2.22 ± 0.03b	233.03 ± 5.48a
Oolong	36.10 ± 2.61a	43.18 ± 3.09 c	1.08 ± 0.08 c	9.97 ± 0.72d	71.90 ± 4.78 c	1.06 ± 0.04d	16.07 ± 1.25 c	145.36 ± 10.11 c	2.02 ± 0.13d	0.56 ± 0.04d	0.44 ± 0.01 f	0.67 ± 0.04 c	3.68 ± 0.19 c	224.47 ± 8.74a
Black	35.72 ± 0.62a	0.00 ± 0.00a	0.42 ± 0.01a	0.29 ± 0.01a	1.09 ± 0.01a	1.05 ± 0.02d	0.73 ± 0.02a	3.53 ± 0.04a	2.24 ± 0.05e	2.80 ± 0.10 f	1.14 ± 0.03 g	3.13 ± 0.11d	9.30 ± 0.28e	133.46 ± 1.86b
Dark	36.28 ± 2.61a	26.11 ± 0.76b	0.99 ± 0.04bc	8.75 ± 0.48 c	79.00 ± 3.77d	0.35 ± 0.01a	18.25 ± 1.01d	135.93 ± 5.78 c	1.22 ± 0.08b	0.19 ± 0.01b	0.22 ± 0.00b	0.36 ± 0.01ab	2.00 ± 0.10b	218.98 ± 4.93a

C, catechin; GCG, (-)-gallocatechin gallate; TF, theaflavin; TF-3-G, theaflavin-3-gallate; TF-3’-G, theaflavin-3’-gallate; TFDG, theaflavin-3,3’-digallate; The total catechins (TCs) content = the total amount of EGC, C, EC, CG, GC, EGCG, GCG, and ECG. The total TFs content = the total amount of TF, TF-3-G, TF-3’-G, and TFDG. Values were expressed as mean ± standard deviation (n = 3). Similar letters represented no significant difference within a column and vice versa (Duncan’s test, P < 0.05).

### Antioxidant activity of six categories of teas in vitro

To demonstrate that different tea possesses varying antioxidant activities, three assays were employed in the present work. In Table 2, green tea exhibited the highest DPPH radical scavenging capacity, followed by yellow tea and white tea, while dark tea exhibited the weakest of all. However, scavenging capacity on ABTS radical was different from the DPPH. Dark tea showed better activity than white tea and black tea. The order of ORAC value was the same as ABTS radical scavenging capacity. Thereinto, green tea showed the highest ORAC value while black tea was significantly lower than the other teas. Owing to single antioxidant index maybe not accurately reflect the total antioxidant activity of each sample. Thus, ACCI was employed to evaluate the total antioxidant capacity. According to Table 2, the property of ACCI in six teas followed the order of green tea, yellow tea, oolong tea, white tea, dark tea, and black tea from high to low.

**Table 2.** Antioxidant activity and antioxidant capacity composite index (ACCI) of the control sample and six categories of teas in vitro

Tea	DPPH (% scavenging rate)	ABTS (% scavenging rate)	ORAC (µmol TE/g·DW)	ACCI
Control	56.06 ± 4.61ab	98.67 ± 1.66 f	81.24 ± 0.71 c	96.10
Green	63.48 ± 3.68b	94.18 ± 2.95e	81.15 ± 0.68 c	98.44
White	57.86 ± 4.99ab	82.85 ± 0.84b	73.56 ± 3.49b	88.57
Yellow	60.59 ± 7.71ab	88.67 ± 0.56d	80.04 ± 2.27 c	94.61
Oolong	56.82 ± 4.87ab	86.41 ± 0.59 cd	78.43 ± 0.53 c	91.21
Black	52.21 ± 4.11a	41.09 ± 1.92a	41.63 ± 2.79a	58.38
Dark	51.18 ± 6.34a	83.86 ± 0.97bc	77.97 ± 0.72 c	87.19

Antioxidant index score = [(sample score/best score) × 100]; ACCI = [(sample score/best score) × 100], averaged for all three assays for each tea for the ACCI. Values were expressed as mean ± standard deviation (n = 3). Similar letters represented no significant difference within a column and vice versa (Duncan’s test, P < 0.05).

### Regulation of lipid metabolism enzymes in vitro

**Figure 2.**
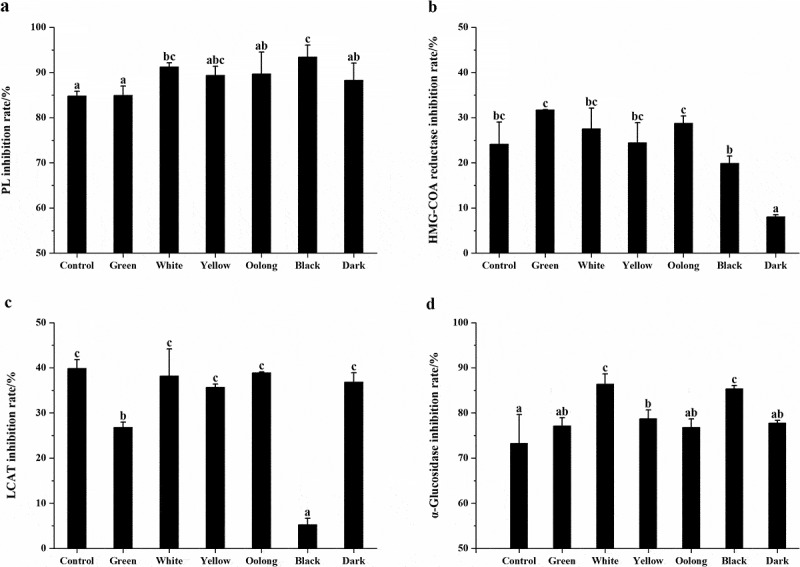
(a) Inhibition rate of PL by the control sample and six categories of teas. (b) Inhibition rate of HMG-COA reductase by the control sample and six categories of teas. (c) Inhibition rate of LCAT by the control sample and six categories of teas. (d) Inhibition rate of α-glucosidase by the control sample and six categories of teas. Values were expressed as mean ± standard deviation (n = 3). Similar letters represented no significant difference in the inhibition rate of each enzyme and vice versa (Duncan’s test, P < 0.05)

Next, we investigated the regulation of PL, HMG-COA reductase, and LCAT activity by tea samples. As shown in Figure 2(a), PL activity was inhibited significantly in response to treated with all tested teas as compared to blank and inhibition rates were all above 80%. Furthermore, PL inhibition capacity was enhanced by increased fermentation degrees in comparison with the control sample. Black tea performed the strongest inhibition activity of PL among the six teas. Therefore, it is probable that the processing methods of these teas altered the concentration and the stability of components involved in the inhibition of PL activity. Besides, all tested teas exhibited inhibitory effects on HMG-COA reductase in Figure 2(b), green tea, white tea, yellow tea, and oolong tea were found to be more effective than the control sample (24%) while black tea and dark tea were less effective than that. Non-fermented green tea and post-fermented dark tea performed the strongest and weakest inhibition of all, respectively, and significant differences were found between them. Tea extracts had no enhancement of LCAT activity in this study, by contrast, it showed significant inhibition of LCAT activity in Figure 2(c). As fresh leaves were processed into six categories of teas, the inhibition rate has decreased in varying degrees. Oolong tea performed the strongest inhibitory effect, while black tea showed the weakest of all.

### Regulation of α-glucosidase activity in vitro

To compare the different potential impact on carbohydrate metabolism of teas, we evaluated the inhibition of α-glucosidase activity by the control sample and six categories of teas. In Figure 2(d), at the same concentration, all teas displayed an inhibitory effect against α-glucosidase and the inhibition capacity of white tea and black tea was significantly higher than that of the control sample. The inhibition rate from high to low was white tea, black tea, yellow tea, dark tea, green tea, and oolong tea with the range from 86.35% to 76.82%.

### Correlations among bioactive components, antioxidant activity, inhibition rate of three lipid metabolism enzymes, and α-glucosidase inhibition rate

Then, we investigated the correlations between chemical components and bioactivities of all tea samples. In Table 3, both TPs and TCs content showed significant positive correlations with antioxidant activity determined by ABTS and ORAC assays (0.955 < r < 0.981, P < 0.01). There was no high correlation between antioxidant activity obtained by DPPH assay and TPs or TCs content, but it was significantly related to the content of CAF (r = 0.772, p < 0.05). Further, significant negative correlations were found between TFs content and antioxidant activity obtained from ABTS and ORAC assays (r = −0.927, r = −0.925, respectively, p < 0.01). Besides, the inhibition rate of PL was positively correlated with TFs content (r = 0.863, p < 0.05) while it was negatively related to TCs content (r = −0.880, p < 0.01). The inhibition rate of LCAT showed significant positive correlations with TPs content (r = 0.902, p < 0.01) and TCs content (r = 0.801, p < 0.05) while it was negatively related to TFs content (r = −0.780, p < 0.05). There was no significant correlation between the inhibition rate of HMG-COA reductase and any other tested bioactive components. Furthermore, the α-glucosidase inhibition rate of teas was positively correlated with TFs content (r = 0.857, P < 0.05) while it was negatively correlated with TCs content (r = −0.823, P < 0.05).

**Table 3.** Pearson’s correlation coefficients among bioactive components, antioxidant activity, inhibition rate of three lipid metabolism enzymes, and α-glucosidase inhibition rate

Item	CAF	TPs	TCs	TFs	DPPH	ABTS	ORAC	PL	HMG-COA reductase	LCAT
TPs	0.533ns									
TCs	0.476 ns	0.925**								
TFs	−0.441ns ns	−0.853*	−0.962**							
DPPH	0.772*	0.572 ns	0.532 ns	−0.354 ns						
ABTS	0.491ns	0.980**	0.981**	−0.927**	0.550 ns					
ORAC	0.521 ns	0.969**	0.955**	−0.925**	0.515 ns	0.983**				
PL	−0.274 ns	−0.711 ns	−0.880**	0.863*	−0.439ns	−0.817*	−0.747 ns			
HMG-COA Reductase	0.340 ns	0.333 ns	0.270 ns	−0.009 ns	0.817*	0.297 ns	0.221 ns	−0.191 ns		
LCAT	0.313 ns	0.902**	0.801*	−0.780*	0.235 ns	0.870*	0. 897**	−0.486 ns	0.086 ns	
α-Glucosidase	−0.114 ns	−0.560 ns	−0.823*	0.857*	−0.191 ns	−0.711 ns	−0.683 ns	0.845*	−0.026 ns	−0.511 ns

CAF, caffeine content; TPs, TPs content; TC, TC content; TFs, total TFs content; DPPH, DPPH radical scavenging rate; ABTS, ABTS radical scavenging rate; ORAC, oxygen radical absorbing capacity; PL, PL inhibition rate; HMG-COA, HMG-COA reductase inhibition rate; LCAT, LCAT inhibition rate; α-Glucosidase, α-glucosidase inhibition rate. ns = non significant and *,** = significant at P < 0.05 or 0.01, respectively.

## Disccusion

In the present study, the results have shown that TPs and catechin components contributed to the antioxidant activity in all categories of teas. In brief, within the six teas made by a single cultivar – – Meizhan, green tea was superior to partially fermented and fully fermented teas in terms of antioxidant activity owing to the relatively high content of catechins, especially for EGCG [33–35]. Hence, it is identified that the total antioxidant activity of teas was not related to a particular kind of polyphenol but the combined activity of diverse antioxidants, including catechins and other polyphenols [36,37]. Furthermore, TFs content gradually increased as tea leaves were fermented and the increase of TFs content was negatively correlated with the TCs levels [38,39]. It might be a reason for significant negative correlations between TFs content and antioxidant activity obtained from ABTS and ORAC assays. The other alternative explanation is that the antioxidant capacity promoted by TFs was not enough to compensate the decrease by TCs [40]. TFs and other oxidation products of EGC and EC were first found in green tea but are widely found in various teas, regardless of their fermentation status [41]. Thus, even if white tea is not fully fermented tea, it could generate and accumulate TFs in processing [42].

Tea intake could decrease serum cholesterol levels in animals and humans through decreasing dietary cholesterol absorption and increasing fecal excretion [43,44]. PL, HMG-COA reductase, and LCAT are the three main lipid metabolism enzymes 10. PL is considered a key enzyme for dietary lipid absorption in humans [45]. In our study, teas with higher TFDG content (black tea and white tea) exhibited better PL activity inhibition than the others, which is identical with the previous reports [46]. The inhibition rate was significantly and highly correlated with TFs content, it suggested that TFs might be the dominant contributor to the inhibition effect of fermented teas [45]. Therefore, the highest TFs content in black tea could be explained that its strongest inhibition capacity against PL activity. Moreover, all tested teas performed an inhibition effect on HMG-COA reductase activity, a rate-limiting enzyme of cholesterol biogenesis [47,48], which might contribute to the cholesterol-lowering effect of tea [49]. The reaction catalyzed by LCAT involves the transfer of an acyl group in the sn-2 site of phosphatidylcholine to the 3-b-hydroxyl group of cholesterol, which yields lysophosphatidylcholine and cholesterol-ester products [50]. The early work showed that tea extracts could promote the activity of LCAT [51],10. Nevertheless, in our study, treatment with tea extracts has been found to decrease LCAT activity in vitro. It is supposed that the lowering of LCAT activity may not contribute to the effect of lipid-lowering caused by tea consumption. The real reason, however, needs to be explored.

α-glucosidase is responsible for the hydrolysis of terminal non-reducing 1,4 linked α-glucose residues leading to the release of absorbable monosaccharides to enter the blood stream [52]. In the present work, all categories of teas displayed an excellent inhibitory effect against α-glucosidase. And the inhibition rate was positively correlated with TFs content while negatively correlated with TCs levels. Previous studies have reported that TFs were better inhibitors of α-glucosidase in comparison to catechins [53]. It means that white and black tea with a relatively higher content of TFs has highlighted the potential of α-glucosidase inhibition. Through the inhibition of α-glucosidase activity, the digestion and absorption of intestinal glucose could be reduced [54], thus, delaying glucose release from dietary carbohydrates is an effective approach for glycemic control [55].

## Conclusion

Our findings verified that the manufacturing process influences the chemical components and bioactivity of teas and also found a significant correlation between them. Green tea displayed the highest antioxidant activity among six categories of teas, which is related to its highest content of TPs. All categories of teas performed remarkable effect on inhibition of α-glucosidase and regulation of the PL, HMG-COA reductase, and LCAT. The potential contributors were TPs and TFs, respectively. This work has the potential to provide a foundation for improving processing methods to strike a balance between chemical compounds and bioactivity and leading us to propose that various categories of tea could possess different profits for human health.

## Highlights

- Six categories of teas were made by single cultivar C. sinensis Meizhan.
- Tea processing methods could affect the content of non-volatile compounds.
- All categories of teas showed strong antioxidant activity and inhibitory of PL, LCAT, HMG-COA reductase, and α-glucosidase activity in vitro
- The potent inhibitors are total catechins and theaflavins
